# The Use of the Reassignment Technique in the Time-Frequency Analysis Applied in VHF-Based Passive Forward Scattering Radar

**DOI:** 10.3390/s20123434

**Published:** 2020-06-17

**Authors:** Marek Płotka, Karol Abratkiewicz, Mateusz Malanowski, Piotr Samczyński, Krzysztof Kulpa

**Affiliations:** Institute of Electronic Systems, Faculty of Electronics and Information Technology, Warsaw University of Technology, 00-665 Warsaw, Poland; k.abratkiewicz@elka.pw.edu.pl (K.A.); M.Malanowski@elka.pw.edu.pl (M.M.); P.Samczynski@elka.pw.edu.pl (P.S.); k.kulpa@elka.pw.edu.pl (K.K.)

**Keywords:** passive forward scattering radar, forward scattering radar, assive radar, radar measurements, time-frequency analysis, time-frequency reassignment

## Abstract

This paper presents the application of the time-frequency (TF) reassignment technique in passive forward scattering radar (FSR) using Digital Video Broadcasting – Terrestrial (DVB-T) transmitters of opportunity operating in the Very High Frequency (VHF) band. The validation of the proposed technique was done using real-life signals collected by the passive radar demonstrator during a measurement campaign. The scenario was chosen to test detection ranges and the capability of estimating the kinematic parameters of a cooperative airborne target in passive FSR geometry. Additionally, in the experiment the possibility of utilizing FSR geometry in foliage penetration conditions taking advantage of the VHF band of a DVB-T illuminator of opportunity was tested. The results presented in this paper show that the concentrated (reassigned) energy distribution of the signal in the TF domain allows a more precise target Doppler rate to be estimated using the Hough transform.

## 1. Introduction

Over the past decades, passive radars have evolved significantly [1,2,3], which can be seen in numerous demonstrations and works devoted to this topic [4,5,6,7,8]. This has resulted from the advantages of passive radars and the possibility to detect targets which do not have their own emission. In fact, issues related to passive radars are generally widely described in the literature, however, there are still problems that require a specific approach.

The main problem in Passive Coherent Location (PCL) radar technology is that using classical passive radar processing for air target detection [9] does not allow one to detect and localize the target in the direction of the illuminator of opportunity, therefore the radar is “blind” at this particular angle and additionally the target is in the first range cell, which provides unclear detection results. This direction is reserved for the collection of the reference signal, which is used for cross-correlation with signals collected from other receiver channels whose measurement antennas are pointed in other surveillance directions where a target echo is suspected to be received. However, this paper deals with the methodology which allows kinematic parameters of the object to be distinguished even if the range information is lost. Various answers to the problem of passive radar direct path “blindness” can be found in numerous literature positions [10,11,12,13,14,15].

A possible solution to this issue may be the employment of reference signal reconstruction [10]. However, this technique only works for digital signals and with sufficient signal-to-noise ratio (SNR) values. Using a beamforming technique to reduce direct signal leakage to surveillance channels [11,12] can also be employed. Aubry et al. [13], used a Constrained Least Squares two-dimensional localization algorithm. Its performance, expressed in terms of Root Mean Square Error (RMSE), is even comparable to square root of the Cramer Rao Lower Bound (CRLB) for some of the simulation scenarios presented. A significant disadvantage of this algorithm is a necessity of employment multiple transmitters of opportunity. A different solution to the localization issue can be found in Aubry et al. [14]. Joint target location is based on a PCL and Time Difference of Arrival (TDOA) measurement techniques. However, the TDOA method requires multiple dislocated radar receivers. Another study on the target location accuracy in multistatic scenario is presented by Anastasio et al. [15].

The other solution for this problem, utilizing a single receiver and single transmitter of opportunity only, might be to additionally use FSR methods in the PCL processing chain. The use of FSR geometry allows passive radar to detect and estimate main movement parameters such as target velocity for the targets crossing the $T_{x}$–$R_{x}$ baseline [16,17]. In such a case, data from the FSR module applied in PCL radars might be used as additional information for the radar tracker, and consequently the detection and velocity estimation from the FSR module to the tracker working in the bistatic range-Doppler plane. Such a method will significantly improve the detection and tracking performance in PCL processing. This fact led the authors to study in more detail the possibility of applying FSR geometry in passive radar, and test novel methods for target Doppler frequency rate estimation which might be applied in PCL processing. An additional motivation was using low-frequency DVB-T sources of illumination in passive radars and their ability to perform foliage penetration. The VHF DVB-T operates in the band of 174–230 MHz [18]. As these are relatively low frequencies, they penetrate the foliage well. The authors did one experiment where a VHF DVB-T based passive radar was deployed in a forest on a low mast around 3 m in height, which was much lower than the surrounding trees, and successfully detected the air targets. The results have been described by Plotka et al. [19]. These valuable results also motivated the authors to check how efficiently the VHF DVB-T illuminator of opportunity would be used in FSR geometry, where the reference signal is also received through the transmitter.

This paper has the following structure: Section 2 presents the passive FSR geometry principle that is considered in this work. Section 3 covers the description of the proposed method for the target Doppler rate estimation. In Section 4, the measurement campaign and the numerical results using the real-life signals are depicted. The paper is closed by comments and conclusions.

## 2. Passive FSR Geometry

**Figure 1:**
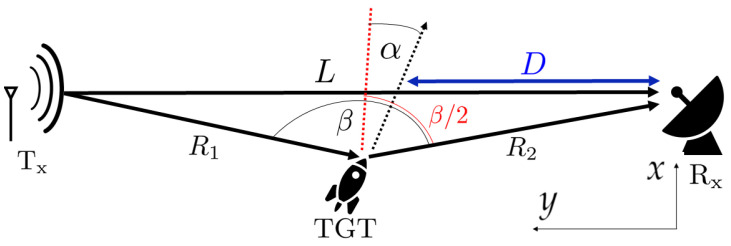
A typical passive forward scattering radar (FSR) geometry. $T_{x}$—transmitter, $R_{x}$—receiver, TGT—target, *L*—baseline, $R_{1}$—range from the transmitter to the target, $R_{2}$—range from the receiver to the target, *D*—range from the receiver to the crossing point, β—bistatic angle, α—angle between the bistatic bisector and the velocity vector.

The forward scattering phenomenon [16,17] is schematically depicted in Figure 1.

In bistatic radars using an electromagnetic wave of a length λ, the Doppler shift $f_{d}$ produced by the target moving at the velocity *V* can be expressed as

(1) $$f_{d}=\frac{2V}{\lambda }\cos\left(\alpha \right)\cos\left(\beta /2\right),$$

where β is the bistatic angle and α is the angle between the bistatic bisector and the movement vector (see Figure 1). For such a spatial configuration where the target crosses the baseline (when the range information is lost and the Doppler frequency tends to 0 Hz) $\beta \approx 180^{\circ }$, thus $f_{d}\approx 0$ Hz, however, the target can be observed in a range $\beta \in (180^{\circ }-\Delta ,180^{\circ }+\Delta )$, where Δ is a small angle, which delivers more information about the target trajectory. In the vicinity of $f_{d}\approx 0$ Hz the signal impinging the receiving antenna can be defined as [20]

(2) $$\phi \left(t\right)=\frac{2\pi }{\lambda }\left[R_{1}\left(t\right)+R_{2}\left(t\right)-L\right].$$

Assuming that the target moves along a linear trajectory at a constant velocity *v* and the velocity vector (composed of $v_{x}$ and $v_{y}$ components corresponding respectively to the x- and y-axis) creates an angle with respect to the *x*-axis that is normal to the baseline $\alpha =\tan^{-1}\left(\frac{v_{y}}{v_{x}}\right)$. If the target crosses the baseline at the point *D* from the receiver (see Figure 1) then $x\left(t\right)=v_{x}t$ and $y\left(t\right)=D+v_{y}t$ which leads to

(3) $$R_{1}\left(t\right)=\sqrt{x{\left(t\right)}^{2}+{\left(L-y(t)\right)}^{2}}$$

and

(4) $$R_{2}\left(t\right)=\sqrt{x{\left(t\right)}^{2}+y{\left(t\right)}^{2}}.$$

Apart from the Doppler history, the target forward radar cross section (FRCS) has a contribution to the signal reaching the receiver in the passive FSR system. Namely, a rectangular target of horizontal $l_{h}$ and vertical $l_{v}$ dimension such that $l_{h}>>\lambda$ and $l_{v}>>\lambda$, is given by [21]

(5) $$\sigma \left(t\right)=\frac{L^{2}}{R_{1}\left(t\right)R_{2}\left(t\right)}\frac{\cos\left(\theta_{T}\left(t\right)-\alpha \right)+\cos\left(\theta_{R}\left(t\right)+\alpha \right)}{2}\Pi \left(\frac{l_{h}}{\lambda }\left(\sin\left(\theta_{T}\left(t\right)-\alpha \right)+\sin\left(\theta_{R}\left(t\right)+\alpha \right)\right)\right)$$

where the target aspect angle with respect to transmitter is $\theta_{T}=\tan^{-1}\left(\frac{x(t)}{L-y(t)}\right)$ and with respect to receiver is $\theta_{R}=\tan^{-1}\left(\frac{x(t)}{y(t)}\right)$ and Π is the function such that $\Pi \left(x\right)=\frac{\sin(x)}{x}$. Then, the signal impinging the FSR receiving antenna can be written as

(6) $$s\left(t\right)=-\sigma \left(t\right)\sin\left(\phi (t)\right).$$

Approximating Equation (6) by the third order Taylor polynomial around the crossing point $t=t_{0}$, Equation (2) becomes

(7) $$\phi \left(t\right)\approx \pi \epsilon {\left(t-t_{0}\right)}^{2}+\pi \zeta {\left(t-t_{0}\right)}^{3},$$

where

(8) $$\epsilon =\frac{v_{x}^{2}}{\lambda }\left[\frac{1}{L-D}+\frac{1}{D}\right],$$

and

(9) $$\zeta =\frac{v_{x}^{2}v_{y}}{\lambda }\left[\frac{1}{{\left(L-D\right)}^{2}}+\frac{1}{D^{2}}\right],$$

and the latter equation goes to 0 for $v_{y}=0$ or D=L/2. Finally, the signal phase can be approximated as follows

(10) $$\phi \left(t\right)\approx \frac{\pi }{\lambda }v_{x}\left(\frac{1}{L-D}+\frac{1}{D}\right){\left(t-t_{0}\right)}^{2},$$

where $v_{x}$ denotes the velocity component which is perpendicular to the baseline *L*, *D* expresses the range from the receiver to the crossing point, and $t_{0}$ is the particular moment the target crosses the baseline. In fact, Equation (10) can be considered as a quadratic phase function resulting in the linear frequency-modulated signal. The modulation factor (also known as a chirp rate, frequency rate, frequency slope, etc.) is valuable information describing the target in the situation when the range measurement is ambiguous, which is the case in passive FSR. Thus, any additional characterization of the target movement is significant in this case. In the literature, different approaches are proposed in order to estimate motion parameters, such as spectrogram analysis [22], chirp rate estimation in the time-frequency (TF) domain [23], or the Radon transform [20]. This paper refers to the latter example, and the authors of this work proposed the method known from the literature to improve the resolution of the TF distribution using the TF reassignment technique in order to distinguish the kinematic parameter of the cooperative target using the Hough transform, which can be interpreted as a discrete realization of the Radon transform [24,25]. The obtained outcomes are compared to existing methodology [20,26,27,28,29], and the improvement in the estimation precision is shown.

## 3. Target Doppler Rate Estimation

Typically, the signal received by the passive FSR antenna after initial processing is presented in the TF domain as a quasi-linear frequency-modulated waveform in accordance to Equation (10). Next, by using the Radon transform the Doppler rate is estimated at the crossing point which determines the kinematic parameters of the target [20,26,28,29]. As shown by Toft et al. [24,25], the Hough transform can be used equivalently as a discrete realization of the Radon method, which may be used for the estimation of the frequency slope of the signal in the TF domain. This approach is very fast and can be implemented in real-life systems, however, some details have to be taken into account. Namely, classical TF representations suffer from the limited resolution resulting from the Heisenberg–Gabor uncertainty principle [30], which spoils the estimation accuracy in this case. Especially when the SNR is low or when several objects cross the baseline at the same time, the method proposed by Ustalli et al. may require additional processing steps. Widely speaking, in many practical applications the short-time Fourier transform (STFT) based approach may be insufficient due to the finite resolution of the TF plane. Additionally, the resolution is strongly dependent on the processing parameters, such as window type, window width, overlap, etc. Even a high-SNR signal can be distributed incorrectly over the TF plane if the processing parameters are badly conditioned. One of the popular and widely applied methods for TF resolution enhancement is TF reassignment [30,31,32,33]. This method can be implemented through the classical STFT-based method, as well as using a recursive version presented by Fourer et al. [34]. The latter is particularly interesting due to the possibility of its efficient implementation and the fast operation of the energy relocation $\left(t,\omega \right)\mapsto \left(\hat{t},\hat{\omega }\right)$. As both the recursive and fast Fourier transform (FFT)-based implementations are equivalent, the FFT-based method is used in this paper as an example of the technique.

In general, the STFT of the signal x(t) can be computed as follows:

(11) $$F_{x}^{h}\left(t,\omega \right)=\int_{R}x\left(\tau \right)h^{*}\left(t-\tau \right)e^{-j\omega \tau }d\tau =M_{x}^{h}\left(t,\omega \right)e^{j\phi_{x}^{h}\left(t,\omega \right)},$$

where $j=\sqrt{-1}$, ${\left(\cdot \right)}^{*}$ is the complex conjugate, R denotes the set of real numbers, $M_{x}^{h}\left(t,\omega \right)$ is the amplitude, and $\phi_{x}^{h}\left(t,\omega \right)$ is the phase of the transform. The energy distribution, commonly called a spectrogram, is defined as a squared absolute value of the STFT and is given by

(12) $$E_{x}^{h}\left(t,\omega \right)={\left|F_{x}^{h}\left(t,\omega \right)\right|}^{2},$$

where |·| denotes the absolute value operator.

Using the relationship defined by Hahn [35], Equation (11) can be transformed into a complex phase as follows:

(13) $$\Phi_{x}^{h}\left(t,\omega \right)=\ln\left(F_{x}^{h}\left(t,\omega \right)\right)=\Lambda_{x}^{h}\left(t,\omega \right)+j\phi_{x}^{h}\left(t,\omega \right),$$

where $\Lambda_{x}^{h}\left(t,\omega \right)=\ln\left(M_{x}^{h}\left(t,\omega \right)\right)$. The concept of complex phase is widely used in the literature as an effective tool for the estimation of signal parameters in the TF domain, which is also applied in the considered approach. For TF reassignment, the relocation operators have to be estimated, which correspond to vectors for both the time and frequency axes along which the energy has to be moved. In the investigated approach, the reassignment operators may be estimated respectively [31]:

(14) $$\hat{t}\left(t,\omega \right)=-ℑ\left(\frac{\partial \Phi_{x}^{h}\left(t,\omega \right)}{\partial \omega }\right)=t-ℜ\left(\frac{\partial \Phi_{x}^{h}\left(t,\omega \right)}{\partial \omega }\right)=t-ℜ\left(\frac{F_{x}^{Th}\left(t,\omega \right)}{F_{x}^{h}\left(t,\omega \right)}\right),$$

(15) $$\hat{\omega }\left(t,\omega \right)=\omega +ℑ\left(\frac{\partial \Phi_{x}^{h}\left(t,\omega \right)}{\partial t}\right)=\omega +ℑ\left(\frac{F_{x}^{Dh}\left(t,\omega \right)}{F_{x}^{h}\left(t,\omega \right)}\right),$$

where *ℜ* is the real and *ℑ* is the imaginary part. $\hat{t}\left(t,\omega \right)$ denotes the relocation along the *t*-axis and $\hat{\omega }\left(t,\omega \right)$ expresses the reassignment vector along the frequency axis. In contrast to another energy concentration technique known from the literature, for example TF synchrosqueezing transform [32], the reassignment method allows strong concentration to be obtained, however, this technique is irreversible. In Equation (14), the expression Th=th(t) is a window multiplied by the linear time ramp with a root in 0, and Dh in Equation (15) denotes the first order derivative of the analyzing window $Dh=\frac{dh(t)}{dt}$. In fact, these operations can be interpreted as follows:

(16) $$\partial F_{x}^{h}\left(t,\omega \right)/\partial t=\int x\left(\tau \right)\frac{\partial h^{*}\left(t-\tau \right)e^{-j\omega (t-\tau )}}{\partial t}d\tau =F_{x}^{Dh}\left(t,\omega \right),$$

as well as:

(17) $$\partial F_{x}^{h}\left(t,\omega \right)/\partial \omega =\int x\left(t-\tau \right)h^{*}\left(\tau \right)\frac{\partial e^{-j\omega \tau }}{\partial \omega }d\tau =-\int x\left(t-\tau \right)h^{*}\left(\tau \right)j\tau e^{-j\omega \tau }d\tau =-jF_{x}^{Th}\left(t,\omega \right),$$

which leads to:

(18) $$\frac{\partial \ln(F_{x}^{h}\left(t,\omega \right))}{\partial t}=\frac{\partial F_{x}^{h}\left(t,\omega \right)}{\partial t}\frac{1}{F_{x}^{h}\left(t,\omega \right)}=\frac{F_{x}^{Dh}\left(t,\omega \right)}{F_{x}^{h}\left(t,\omega \right)}$$

and

(19) $$\frac{\partial \ln(F_{x}^{h}\left(t,\omega \right))}{\partial \omega }=\frac{\partial F_{x}^{h}\left(t,\omega \right)}{\partial \omega }\frac{1}{F_{x}^{h}\left(t,\omega \right)}=-j\frac{F_{x}^{Th}\left(t,\omega \right)}{F_{x}^{h}\left(t,\omega \right)}$$

which can be directly applied in Equations (14) and (15). This means that the reassignment operators can be easily computed through the STFT method using the modified analyzing window, which increases the utility of the method and reduces the computational effort. Equivalently, the method may be implemented using a recursive filter bank, as described in [34].

Finally, the energy relocation using the reassignment method can be expressed as [31]

(20) $$R_{x}^{h}\left(t,\omega \right)={\;\int \int \;}_{R^{2}}{\left|F_{x}^{h}\left(t,\omega \right)\right|}^{2}\delta \left(t-\hat{t}\left(t,\omega \right)\right)\delta \left(\omega -\hat{\omega }\left(t,\omega \right)\right)dtd\omega ,$$

where δ(·) denotes the Dirac distribution. The distribution given by Equation (20) results in strongly concentrated energy on the TF plane, with an enhanced readability and separated components. In fact, the reassignment method usually does not relocate the maximum of the energy but only attracts the surrounding distribution, hence the signal localization remains stable whilst the readability of the transform is improved.

The TF reassignment is a widely used technique in many applications, e.g., ultrasound signal processing [36], audio signal analysis [37,38], or sonar applications [39]. However, despite the high potential of this method, it is still not very popular in the radar community. Namely, in the literature one can find results for the energy concentration in micro-Doppler signature analysis [40,41,42], characterization of frequency shift keying (FSK) signals [43], improving the quality of inverse synthetic aperture radar (ISAR) imaging [44] as well as in direction of arrival estimation [45] and Doppler radar tomography imaging [46]. The novelty presented in this paper is to apply the TF reassignment method and combine it with the Hough transform that aims to extract the Doppler rate of the target with the improved accuracy comparing the classical method existing in the literature.

The valuable properties of the TF reassignment method prompted the authors to apply this technique to analyze radar signals in a passive FSR application. The “energy gathering” properties applied in such an application may improve the accuracy of the Doppler rate estimation in passive FSR systems. This, in fact is the novelty proposed in this paper, since to the authors knowledge there is no similar application that uses the TF reassignment method to improve the accuracy of the Doppler rate estimate in passive FSR system. Additionally, the outcomes are compared to the classical approach existing in the literature with particular emphasis on the usability in real-life data processing which is investigated in the next section.

## 4. Numerical Experiments

### 4.1. Measurement Campaign

**Figure 2:**
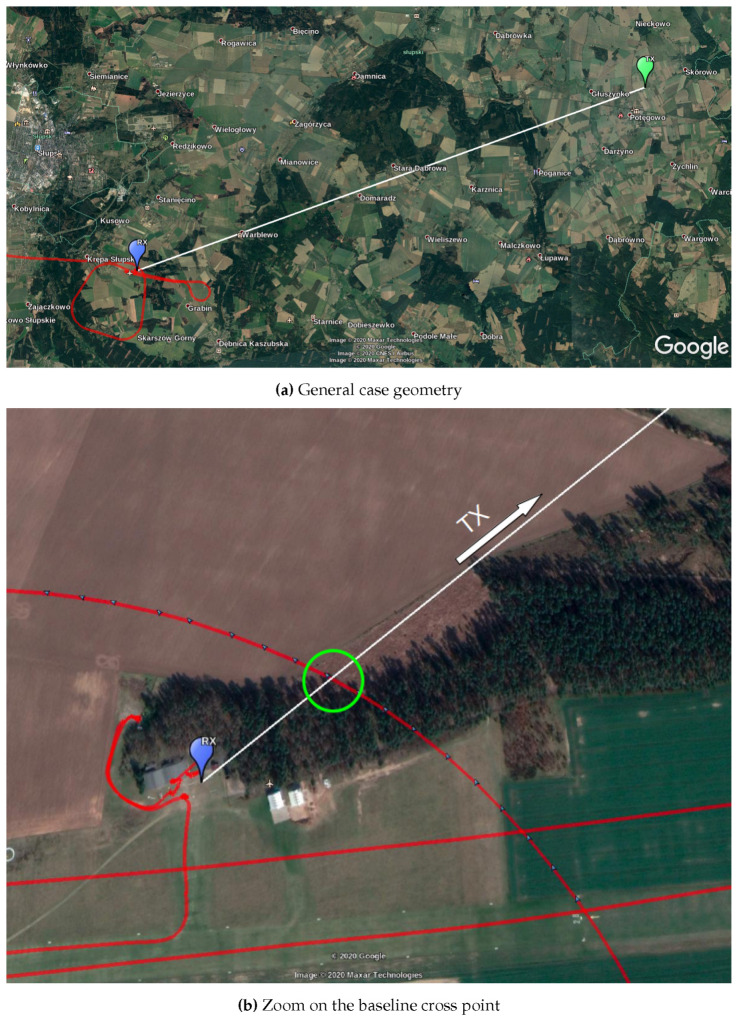
Scenario geometry: transmitter–receiver baseline (white line) cross point marked by a green circle, aircraft trajectory in red.

The measurements took place during the APART GAS 2019 (Active PAssive Radar Trials Ground based, Airborne, Sea-borne) trials. The trials were described by Plotka et al. [19], nevertheless some details of the measurement FSR scenario geometry will be examined here. The positions of the receiving station, transmitter of opportunity, aircraft trajectory, and transmitter–receiver baselines are depicted in Figure 2.

**Figure 3:**
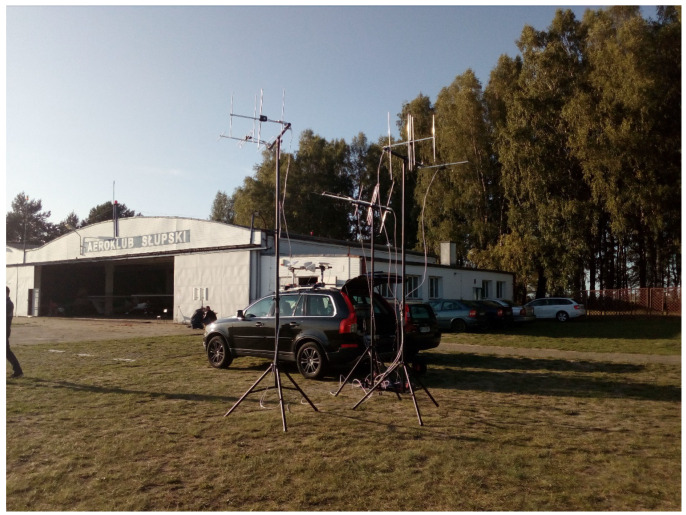
Radar demonstrator during measurements.

During the passive FSR measurements, the radar receiving station was placed in an open space, on an airfield (see Figure 3). The location was chosen to also test FSR geometry in foliage penetration conditions. The receiver was placed close to the forest line, where the trees were in the direction of the transmitter of opportunity—see Figure 2b. The radar demonstrator was equipped with 6 antennas, but only two of them were used in the presented FSR experiment. During the trials the signal from both V- and H-polarized receiving antennas was gathered, however, due to the lack of significant differences between the results only the selected pair of the receiving channels was analyzed. Hence, in the further part of this paper the signal from V-polarized antennas mounted on a tripod mast at the height of ca. 3 m above the ground is presented. The parameters of the employed transmitter of opportunity are listed in Table 1.

**Figure 4:**
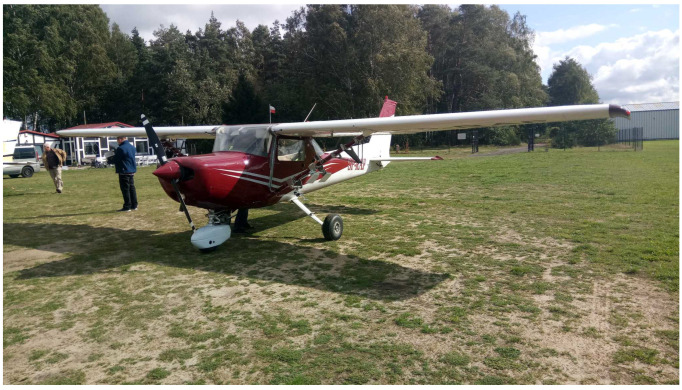
The cooperative aerial target.

During the measurements a cooperative target was used—a light Cessna aircraft (see Figure 4). At the moment of crossing the transmitter–receiver baseline, the aircraft flight parameters were as follows: the target altitude was 196 m above terrain level and velocity was 44 m/s. The distance between receiver and target was 280 m, the distance between transmitter and target was 27.5 km, and the distance between receiver and transmitter was 27.706 km.

**Figure 5:**
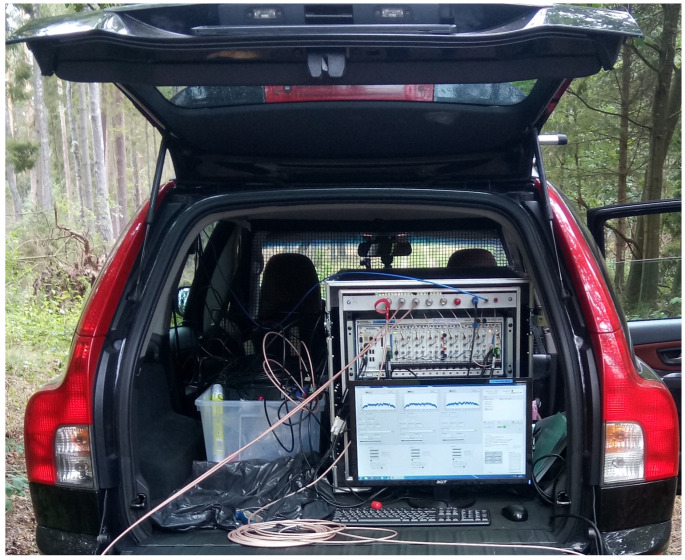
Digital multichannel signal recorder.

The composition of the receiving station was as follows. Antennas: commercial-off-the-shelf (COTS) 4-element Uda-Yagi, with directivity from 6 dBi to 8 dBi, operating in an upper VHF frequency band (170 MHz up to 230 MHz). Analog front-end: COTS channel amplifier, operating in 87–230 MHz frequency band, with 25 dB gain. Digital signal recorder (see Figure 5): Vector Signal Analyzer (VSA) based on National Instruments PXIe components, with six independent input channels synchronized coherently with GPS signal, operating in the frequency range 10 MHz–6.6 GHz with the maximum bandwidth of 50 MHz. More detailed description, of the radar demonstrator hardware, has been presented by Plotka et al. [19].

### 4.2. Results

**Figure 6:**
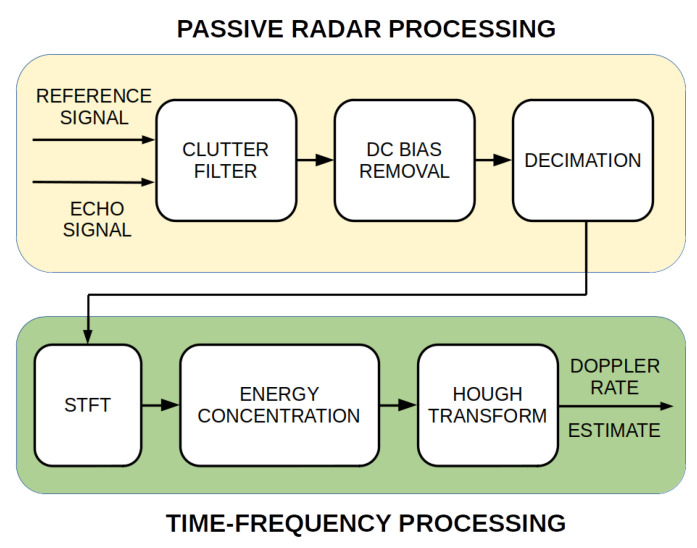
Passive FSR signal processing scheme.

Signals recorded by the passive radar demonstrator were processed with the passive FSR signal processing chain as presented in Figure 6.

At first, signals from two channels (reference and surveillance) were selected for further calculations. All further computations were performed on the signals’ blocks with a 200 ms integration period (which resulted in a velocity resolution equal to 8.13 m/s). Next, a clutter filter was used for removing the reference signal from the surveillance signal [9]. This operation normally significantly reduces target echo power when reaching zero Doppler velocity. In order to limit the scale of this phenomenon, clutter removal filter coefficients were fixed for the time when the target was crossing the baseline [47]. An additional step of signal processing was the removal of direct current (DC) offset, which was achieved by subtracting the mean value from the signal after clutter filtering. The last step of the processing was a multi-stage decimation. According to the simulations carried out, the expected Doppler frequency was not greater than 100 Hz. The input sampling rate of the recorded signals was equal to 8 MHz, so the filtered signal might have been down-sampled a few thousand times without losing valuable information. This step considerably reduced the number of unnecessary subsequent calculations. It should be mentioned that the bistatic range resolution for the processed signal is equal to 37.5 m (this is the size of the first range cell).

**Figure 7:**
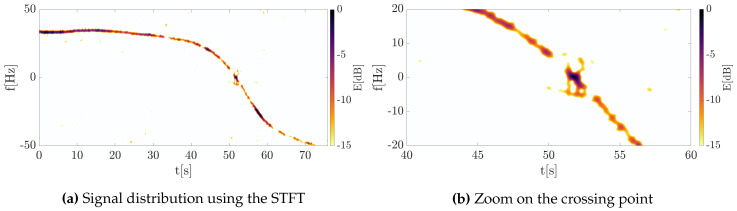
The energy distributions of the measured signal obtained using the classical short-time Fourier transform (STFT).

Next, the signal was transformed into the TF domain using Equation (11) giving the classical signal distributions on the 2D TF plane, as shown in Figure 7. The Doppler history is clearly visible for the entire trajectory, however, some interference related to multipath propagation and clutter removal algorithm are apparent, especially at the point when the waveform changes the frequency sign. Additionally, the clutter cannot be suppressed at this point due to the presence of the useful signal that should not be filtered out.

**Figure 8:**
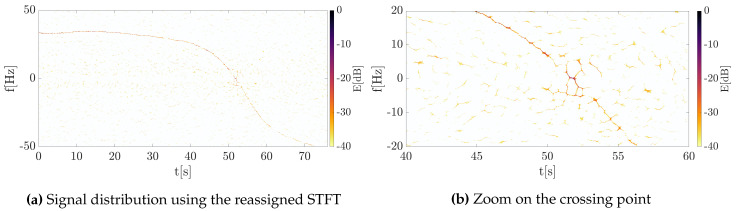
The energy distributions of the measured signal obtained using the concentrated STFT.

The same signal was processed using the concept of energy concentration. The outcomes for this approach are depicted in Figure 8, where the significant concentration was obtained. In such a case, the component extraction and separation are obtainable even in the case of low SNR.

**Figure 9:**
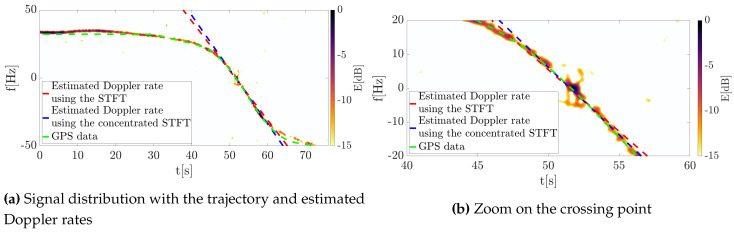
Results of the Hough transform obtained for the two methods investigated—the classical and the reassigned STFT.

Both classical and reassigned distributions were obtained using an 8192-point FFT and the Gaussian window of a standard deviation σ=0.2. The shift of the window in consecutive steps the processing was equal to 1 sample in order to provide precise signal representation. Then, in accordance to the approach proposed by Ustalli et al. [20,26,28,29], the Hough transform was applied to estimate the signal Doppler rate as a straight line on the TF plane composed by the Doppler history of the signal near the zero-intersection point. The results are depicted in Figure 9.

As can be observed, both the classical and the concentrated distributions allowed the Doppler rate to be estimated. The selected sections of both distributions $f\in \left(-20,20\right)$ Hz, $t\in \left(40,60\right)$ s containing the most important parts of the signal were processed using the Hough transform, which gave results corresponding to the frequency slope at the interesting point. These values were estimated as follows: $f_{S}=-3.6392\frac{\mathrm{Hz}}{s}$ for the classical STFT distribution and $f_{R}=-3.9873\frac{\mathrm{Hz}}{s}$ for the reassigned spectrogram.

**Figure 10:**
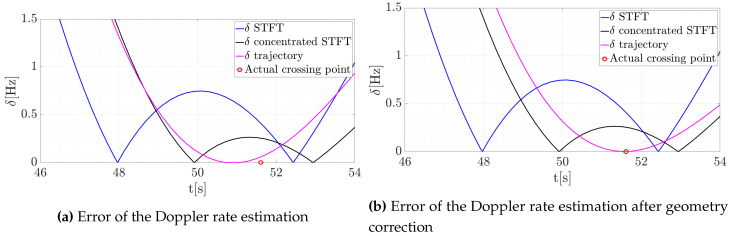
Absolute value of estimated errors of the Doppler rate estimation—initial error and after correction.

Both estimated lines coincide with the Doppler history, and the visual analysis of them does not give an answer as to which of them is more precise. Thus, in order to verify the correctness of the estimate, an additional line was created. The Hough transform was applied on the curve fragment derived from GPS data (see the green line in Figure 9), allowing the precise Doppler rate to be assessed. Next, the error between the reference (GPS data) and two estimated lines was computed. However, due to the limited precision of the DVB-T transmitter localization, as well as the smoothing of the GPS-based trajectory, the actual crossing point is mismatched. The additional sources of mismatch error may be connected with the GPS logger. This device’s coordinates estimation accuracy is limited, and as well as its own location inside the aircraft also mattered. An another point which had an impact on the accuracy is the information about the transmitter ($T_{x}$) position. For the analysis, the authors took the $T_{x}$ position from an open database and validated the $T_{x}$ coordinates using Google Maps. However, the accuracy of the $T_{x}$ position is also often given with a precision of several meters, which might have had an impact on the presented results. Therefore, an additional simulation was performed in which the geometry was appropriately modified by changing the transmitter position that aims to reduce this fault. After these modifications the error was eliminated, and the results for both the initial and modified trajectories are depicted in Figure 10. The plots show the absolute value of the estimation error δ.

The proposed method allowing signal concentration in the TF to be obtained improved the precision of the Doppler rate estimation. As can be observed, the error was reduced for both parameters: the Doppler rate, and for the time when the target crossed the baseline. This result indicates the effectiveness of the proposed approach.

For the Hough transform, the computational complexity increases at a rate of $O\left(A^{m-2}\right)$ where *A* denotes the size of the image space and *m* corresponds to the number of parameters applied in the processing pipeline. In the proposed method, the Hough transform is the same as in the classical approach with STFT. Therefore, the only difference results from the signal processing associated with the reassignment operation. For this reason, only the STFT and the reassigned STFT computational complexity are compared in Table 2 [40].

The increase in computational complexity in the concentrated spectrogram technique results from the fact that 3 distributions are necessary to be implemented in accordance to Equation (20). Namely, the first distribution is the classical STFT with the original window according to Equation (11). The second one corresponds to Equation (16), and the last one to Equation (17). Hence, the precision of the Doppler rate can be improved at the expense of computational complexity. In fact, the processing time may be reduced through the manipulation of the processing parameters. For the purpose of this paper, the number of frequency bins and the window shift were assumed with some redundancy for high resolution of distributions. In practice, these parameters can be reduced to ensure fast processing. For the parameters defined above, the computation time for the spectrogram ($t_{S}$) and the reassigned spectrogram ($t_{R}$) was respectively $t_{S}=0.42$ s and $t_{R}=1641.09$ s, but for 1024 points of the FFT this time was reduced to $t_{S}=0.17$ s and $t_{R}=90.73$ s. For the purposes of calculations, a computer with an Intel i7-7700HQ 2.8 GHz processor, 16 GB DDR4 RAM, an SSD hard drive, and a 64-bit Windows 10 system was used. The calculations were performed in the Matlab environment. Consequently, by reducing the distribution quality and for the window shift H=2, the processing time was additionally decreased to $t_{S}=0.06$ s and $t_{R}=29.62$ s with nearly the same readability of the distribution. This analysis shows how the processing time may be easily manipulated and reduced with almost conserved resolution, allowing enhanced estimation of the motion parameters to be performed.

## 5. Discussion and Conclusions

In this paper, the concept of applying the reassignment technique in passive FSR applications has been proposed. The main purpose of using this method was to enhance the readability of the energy distribution in the TF domain, which improved the result of the Hough transform and finally the precision of the Doppler rate estimation in the passive FSR system. In the considered case, the passive FSR system used a DVB-T VHF signal as a source of illumination, and the cooperating target (Cessna aircraft) crossed the baseline between the signal transmitter and the passive radar demonstrator. The investigation showed that the low-frequency illuminating signal allows detection in the specific geometry to be carried out and, additionally, by using the concept of energy concentration the signal processing pipeline may be improved. Such an improved energy representation can be utilized in further processing for different purposes, such as the estimation of the maneuvering target trajectory for the passive FSR configuration where the range information is ambiguous and the only data describing the target is its Doppler rate related to the velocity and the trajectory. The concept of using passive FSR systems may be implemented in various real-life systems known from the literature as well as in completely new applications. The authors consider realization of the technique in such applications:

- Border surveillance—the system may prevent to illegal transport and emigration outside the border crossing. In such a case, the illuminator of opportunity can be located both, in the same region as the receiver and in a foreign terrain which expands the possibility of the systems. In addition, passive FSR with other radars with rotating antenna as a source of illumination can be used in such scenario to detect and localize slow moving targets, which can be an extension of work described by Raja Abdullah et al. [48], where a cooperative transmitter was employed as an illuminator of opportunity.
- Debris detection—a passive FSR system can detect and estimate movement parameters of debris which may be of particular importance for space applications. In that case, the baseline can be established between a stationary receiver and the transmitter in the orbit working with one of the popular radiocommunication systems (Starlink, GPS, etc.). Such configuration may allow fast and precise debris detection in order to prevent other satellites destruction [49,50].
- Airport runway—the problem of airport runway security arises simultaneously with technology related to drones production and development. The passive FSR system may be deployed at the airport and detect non-cooperating objects appearing in the flight path of the aircraft. The system can be particularly useful for facilities not equipped with a transponder. In order not to employ another signal transmitter, an Airport Surveillance Radar with a rotating antenna may be used, which is routinely available at most airports [48].
- Ground aerial monitoring—where traffic is prohibited or where the velocity is strictly limited the passive FSR system and especially the algorithm proposed in this paper may be useful. The Doppler rate estimate may be an efficient solution for regions in which the velocity of cars has to be restricted and precisely monitored.
- Intruder detection—another application of the system in question is its usage in security systems for restricted areas, e.g., military or governmental areas. Implementation of the passive radar receiver in such a terrain when surrounding commercial transmitters (e.g., DVB-T) may improve detection capabilities and increase security.

In the future, the authors intend to work on the above applications and their implementation in passive FSR systems. An additional perspective is to investigate the possibility of applying real-time processing for the methods presented in this paper.

## Figures and Tables

**Table 1:** Main parameters of the transmitter of opportunity.

Name	“Lębork Skórowo Nowe”
Distance to receiver	27.8 km
Location height	96 m a.m.s.l
Mast height	93 m
EIRP	10.4 kW
Frequency	184.5 MHz
Signal bandwidth	7 MHz
Polarization	Vertical

**Table 2:** Computational complexity for the STFT and the reassigned STFT in the O notation. *N*—amount of points in the FFT analysis, K=⌈M/H⌉—amount of time instants for which the FFT has to be applied for *M*—signal length in samples, and *H*—window shift in samples.

	STFT	Reassigned STFT
Computational complexity	$O\left(KN\log_{2}\left(N\right)\right)$	$3\cdot O\left(KN\log_{2}\left(N\right)\right)$
